# Allitridi Inhibits Multiple Cardiac Potassium Channels Expressed in HEK 293 Cells

**DOI:** 10.1371/journal.pone.0051550

**Published:** 2012-12-14

**Authors:** Xiao-Hui Xu, Hai-Ying Sun, Yan-Hui Zhang, Wei Wu, Kui-Hao Chen, Yi Liu, Chun-Yu Deng, Xi-Yong Yu, Man-Wen Jin, Gui-Rong Li

**Affiliations:** 1 Department of Pharmacology, Tongji Medical College, Huazhong University of Science and Technology, Wuhan, China; 2 Department of Medicine, Li Ka Shing Faculty of Medicine, University of Hong Kong, Hong Kong, China; 3 Research Centre, Guangdong General Hospital, Guangzhou, China; 4 Department of Physiology, Li Ka Shing Faculty of Medicine, University of Hong Kong, Hong Kong, China; Temple University, United States of America

## Abstract

Allitridi (diallyl trisulfide) is an active compound (volatile oil) from garlic. The previous studies reported that allitridi had anti-arrhythmic effect. The potential ionic mechanisms are, however, not understood. The present study was designed to determine the effects of allitridi on cardiac potassium channels expressed in HEK 293 cells using a whole-cell patch voltage-clamp technique and mutagenesis. It was found that allitridi inhibited hKv4.3 channels (IC_50_ = 11.4 µM) by binding to the open channel, shifting availability potential to hyperpolarization, and accelerating closed-state inactivation of the channel. The hKv4.3 mutants T366A, T367A, V392A, and I395A showed a reduced response to allitridi with IC_50_s of 35.5 µM, 44.7 µM, 23.7 µM, and 42.4 µM. In addition, allitridi decreased hKv1.5, hERG, hKCNQ1/hKCNE1 channels stably expressed in HEK 293 cells with IC_50_s of 40.2 µM, 19.6 µM and 17.7 µM. However, it slightly inhibited hKir2.1 current (100 µM, inhibited by 9.8% at −120 mV). Our results demonstrate for the first time that allitridi preferably blocks hKv4.3 current by binding to the open channel at T366 and T367 of P-loop helix, and at V392 and I395 of S6 domain. It has a weak inhibition of hKv1.5, hERG, and hKCNQ1/hKCNE1 currents. These effects may account for its anti-arrhythmic effect observed in experimental animal models.

## Introduction

Garlic (*Allium sativum* L.) and its constituents have been reported to have multiple beneficial effects including anti-microbial effects [1], anti-cancer [2], [3], [4], [5], lowering blood pressure [6], [7], cardiac protection against ischemia/reperfusion insult [8], [9], reducing serum cholesterol [2], inhibiting angiogenesis [5], [7], enhancing thrombolysis [10], and also anti-arrhythmic effect [11]. However, the mechanisms underlying these beneficial effects are not fully understood, and the ionic mechanism of garlic constituents for anti-arrhythmic effect is unclear.

A recent report demonstrated that allitridi selectively inhibited the transient outward potassium current I_to_ and had no significant effect on the ultra-rapidly delayed rectifier potassium current I_Kur_ and L-type calcium current (I_Ca.L_) in human atrial myocytes [12]. However, the anti-arrhythmic effect and the prolongation of cardiac action potential duration and effective refractory period reported previously with allitridi and/or garlic constituents [11], [13], [14] can not fully interpreted by the inhibition of cardiac I_to_, because the 4-aminopyridine-sensitive I_to_ was not expressed in cardiac myocytes in some species (e.g. guinea pigs and pigs) [15], [16]. On the other hand, it is unknown the molecular determinants of allitridi for inhibiting cardiac I_to_. The present study was therefore designed to determine the molecular determinants of allitridi for blocking Kv4.3 channels (coding human cardiac I_to_) [17], and to investigate whether allitridi would inhibit other cardiac potassium channels stably expressed in HEK 293 cells, including hERG (coding human cardiac I_Kr_, rapidly-delayed rectifier potassium current) [18], hKCNQ1/hKCNE1 (coding human cardiac I_Ks_, slowly-delayed rectifier potassium current) [19], hKv1.5 (coding human cardiac I_Kur_) [20], and hKir2.1 channels (coding human cardiac I_K1_, inward rectifier potassium current) [21] stably expressed in HEK 293 cells using mutagenesis and whole-cell patch voltage-clamp techniques. Our results demonstrated that allitridi preferably blocked hKv4.3 channels by interaction with the sites of P-loop helix and S6 domain of the channel, and it also inhibited hKv1.5 channels, hERG channels, and hKCNQ1/hKCNE1 channels expressed in HEK 293 cells with a relatively weak effect.

## Materials and Methods

### Cell Culture, Mutagenesis and Gene Transfection

The established HEK 293 cell lines stably expressing hKv4.3 (*KCND3*) [22], hKv1.5 (*KCN5A*) [23], [24], hERG (Kv11.1 or *KNCH2*) [25], hKCNQ1/hKCNE1 [26], and hKir2.1 [27], respectively, were cultured in Dulbecco’s modified eagle’s medium (DMEM, Invitrogen, Hong Kong) supplemented with 10% fetal bovine serum and corresponding selective antibiotics. The hKv4.3 channel mutants, T366A, T367A, V392A, I395A and V399A generated using the QuickChange Site-directed Mutagenesis kit (Stratagene, La Jolla, CA, USA), were transiently expressed in HEK 293 cells with a 36-mm culture dish using 10 µl of Lipofectamine 2000 with 4 µg of hKv4.3 mutant cDNA in pcDNA3 vector. The point mutants were confirmed with DNA sequencing. Cells used for electrophysiology were seeded on a glass cover slip.

### Solutions and Chemicals

Tyrode solution contained (mM) NaCl 140, KCl 5.4, MgCl_2_ 1.0, CaCl_2_ 1.8, NaH_2_PO_4_ 0.33, 4-(2-hydroxyethyl)-1-piperazineethanesulfonic acid (HEPES) 10.0 and glucose 10 (pH adjusted to 7.3 with NaOH). The pipette solution contained (mM) KCl 20, K-aspartate 110, MgCl_2_ 1.0, HEPES 10, ethyleneglycoltetraacetic acid (EGTA) 5, GTP 0.1, Na- phosphocreatine 5, and Mg-ATP 5 (pH adjusted to 7.2 with KOH). Allitridi was obtained from Qingjiang Pharmaceutical (Nanjing, Jiangshu, China). All other reagents were obtained from Sigma-Aldrich (St. Louis, MO).

### Electrophysiology

Cells on a coverslip were transferred to an open cell chamber (0.5 ml) mounted on the stage of an inverted microscope and superfused with Tyrode solution at ∼2 ml/min. The whole cell patch-clamp technique was used as described previously [22]–[27]. The whole-cell membrane currents were measured using an EPC-10 amplifier and Pulse software (Heka Elektronik, Lambrecht, Germany). Borosilicate glass electrodes (1.2-mm OD) were pulled with a Brown/Flaming puller (model P-97, Sutter Instrument, Nato, CA) and had resistances of 2–3 MΩ when filled with the pipette solution. A 3-M KCl agar bridge was used as the reference electrode. The tip potential was zeroed before the patch pipette contacted the cell. After the giga-Ohm seal was obtained, the cell membrane was ruptured by applying gentle pressure to establish a whole-cell configuration. Series resistance (Rs) was 4–6 MΩ and was compensated by 50–70% to minimize voltage errors. The liquid junction potential (14.7 mV) calculated with the software Clampex was not corrected in the experiment and data analysis. Cell membrane capacitive transient was electrically compensated with the Pulse software. Current and voltage signals were low-pass filtered at 5 kHz and stored in the hard disk of an IBM compatible computer. All experiments were conducted at room temperature (22–23°C).

### Statistical Analysis

The data are expressed as mean±SEM. Paired and/or unpaired Student’s t-test were used as appropriate to evaluate the statistical significance of differences between two group means, and ANOVA was used for multiple groups. Values of P<0.05 were considered to be statistically significant.

## Results

### Inhibition of hKv4.3 Current by Allitridi

**Figure 1:**
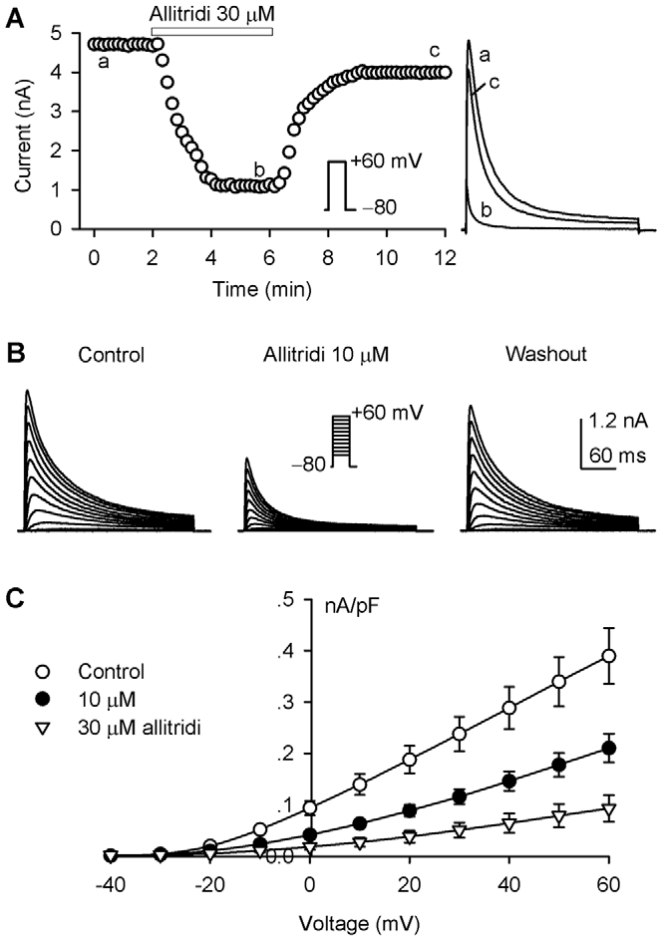
Effect of allitridi on hKv4.3 current. **A.** Time course of hKv4.3 step current recorded in a representative HEK 293 cell stably expressing *KCND3* gene in the absence and presence of 30 µM allitridi with a 300-ms test pulse from –80 to +60 mV (inset). Original current traces at corresponding time points are shown in right side of the panel. **B.** Voltage-dependent hKv4.3 current traces recorded in another cell using the protocol as shown in the inset in the absence and presence of 10 µM allitridi. **C.** Current-voltage (*I-V*) relationships of hKv4.3 current in the absence and presence of 10 and 30 µM allitridi (n = 18, P<0.05 or P<0.01 vs. control at −10 to +60 mV).

The previous study reported that the IC_50_ of allitridi for inhibiting human atrial I_to_ was about 45 µM [12]. Here we initially used a concentration of 30 µM allitridi to determine the potential inhibition of hKv4.3 current stably expressed in HEK 293 cells. Considering that allitridi is a volatile sulfate compound [28], the experimental working bath solution with 30 µM allitridi was immediately prepared before the drug application. The hKv4.3 current recorded with 300-ms voltage step to +60 mV from a holding potential of −80 mV was rapidly inhibited by 30 µM allitridi. The inhibitory effect reached a steady-state level within 130 s application, and was gradually reversed by washout (Fig. 1A). Significant inhibition of voltage-dependent hKv4.3 current could also be observed with 10 µM allitridi (Fig. 1B).

Figure 1C illustrates the current-voltage relationships of mean values of hKv4.3 current in the absence and presence of 10 and 30 µM allitridi. Allitridi significantly inhibited the current at test potentials of −10 to +60 mV (n = 16, P<0.05 or P<0.01 vs. control). It is interesting to note that the current (at +60 mV) was inhibited by 46% and 78% with 10 and 30 µM allitridi and the inhibitory efficacy of hKv4.3 current by allitridi is much stronger than that in human atrial I_to_ reported previously [12]. We also observed a weak blocking effect of hKv4.3 current with a pre-prepared allitridi working solution (40–50% inhibition with 30 µM allitridi, data not shown). The different efficacy suggests that the immediate preparation of experimental working bath solution is crucial for obtaining the accurate pharmacological profile of this volatile compound.

**Figure 2:**
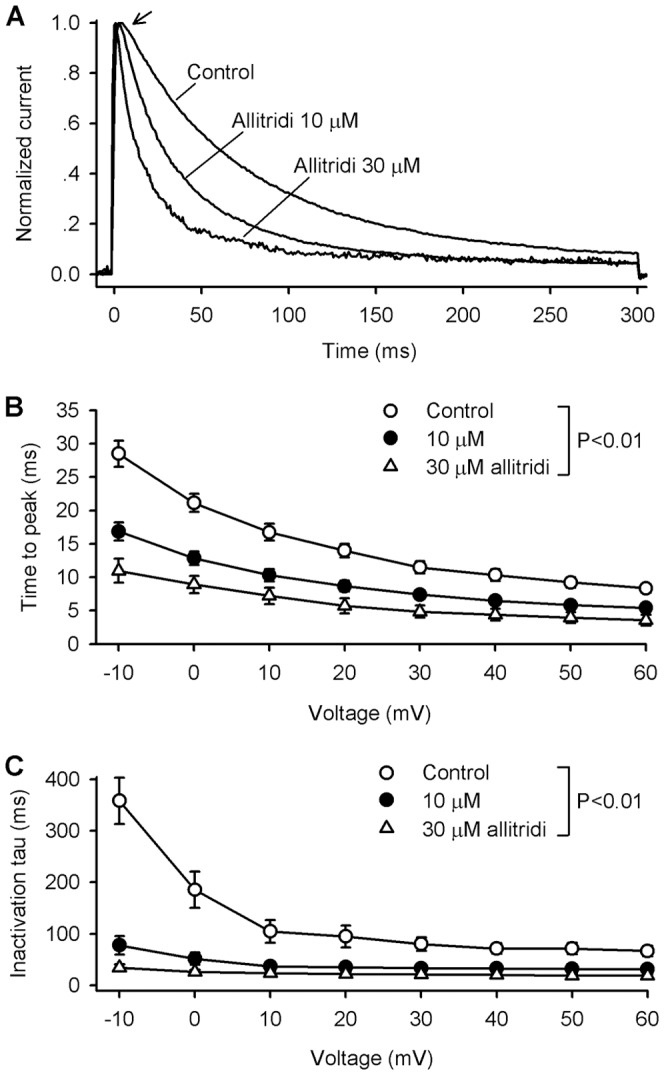
Open channel blockade of hKv4.3 by allitridi. **A.** Normalized current (+60 mV) in a representative cell before (control) and after 10 and 30 µM allitridi. The arrow indicates the changes of the time to peak of the current activation. **B.** Mean values of the time to peak of the current activation at −10 to +60 mV before and after application of 10 and 30 µM allitridi (n = 11 experiments, P<0.01 vs. control). **C.** Mean values of time constant of hKv4.3 current inactivation at −10 to +60 mV before and after application of 10 and 30 µM allitridi (n = 11 experiments, P<0.01 vs. control).

In addition to the reduction of current amplitude, allitridi induced a facilitation of hKv4.3 current inactivation (Fig. 1A and 1B), this suggests that allitridi may block the open channels. To demonstrate the open channel blocking properties, we normalized the current traces, measured the time to peak of hKv4.3 current, and fitted the inactivation process with a mono-exponential equation in the absence and presence of 10 and 30 µM allitridi. The normalized current at +60 mV showed a quick inactivation and a reduced time to peak of current activation in a representative cell (Fig. 2A). Figure 2B and 2C illustrate the mean values of the time to peak of activation and the time constant (tau) of inactivation. The time to peak of hKv4.3 activation and the time constant of hKv4.3 current inactivation were significantly reduced at all test potentials (−10 to +60 mV) by 10 and 30 µM allitridi (P<0.01 vs. control). The acceleration of the activation (time to peak) and the inactivation time constant indicates that allitridi inhibits hKv4.3 current by blocking the open channel.

**Figure 3:**
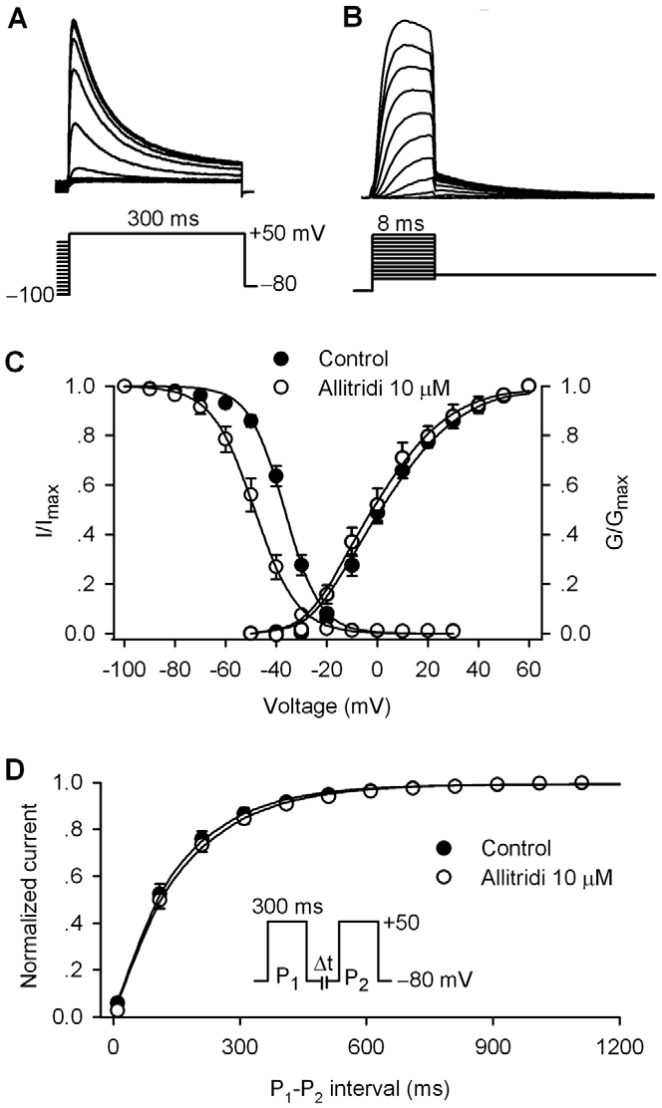
Effect of allitridi on kinetics of hKv4.3 current. **A.** Protocol and current traces used to assess availability (I/I_max_, steady-state inactivation) of hKv4.3 current. **B.** Protocol and tail current traces used to assess activation conductance (G/G_max_, steady-state activation) of hKv4.3 current. **C.** Mean values of hKv4.3 current (I/I_max_) variables and conductance (G/G_max_) variables before and after 10 µM allitridi were fitted to the Boltzmann function: g = 1/(1+exp((V_1/2_-V_t_)/K)), where V_1/2_ is the voltage of 50% channel availability or maximal activation of the channel, V_t_ is the test potential, and K is slope factor. **C.** Mean values of recovery time course of hKv4.3 current from inactivation, determined with protocol as shown in the inset before and after 10 µM allitridi, were fitted to a mono-exponential function.

Figure 3 displays the effect of allitridi on kinetics of hKv4.3 current. Figure 3A and 3B shows the representative current and voltage protocol used for determining the availability of hKv4.3 current and the activation with tail current. Figure 3C illustrates the mean values of the variables of availability (I/I_max_) of hKv4.3 current using the protocol as shown in Fig. 3A and the variables of activation conductance (G/G_max_) measured from the current tail as shown in Fig. 3B in absence and presence of 10 µM. The variables of I/I_max_ and G/G_max_ were fitted to a Boltzmann function in individual cells as described previously [29]. The V_1/2_ of hKv4.3 channnl availability was negatively shifted by 11.9 mV (from −36.7±1.1 mV in control to −48.6±0.9 mV in 10 µM allitridi, n = 12, P<0.01 vs. control), while the V_1/2_ of activation conductance of the current was not altered (3.1±1.2 mV in control, −0.2±1.5 mV in allitridi, n = 10, P = NS vs. control).

Figure 3D shows the mean values of recovery time course of hKv4.3 current from inactivation determined by a paired pulse using a 300-ms step to +50 mV from a holding potential of −80 mV with variable P_1_–P_2_ interval as shown in the inset. The recovery time course was fitted to a mono-exponential function in individual cells before and after 10 µM allitridi application. The recovery time constant (τ) was 132.1±4.1 ms in control, and 126.7±5.1 ms in 10 µM allitridi (n = 10, P = NS vs. control). In another group of experiments, we found that inhibition of hKv4.3 current by allitridi (10 µM) was use- or rate-independent from 0.2 Hz to 3.3 Hz (n = 6, data not shown). The results suggest that allitridi has no effect on the recovery of hKv4.3 channels from inactivation, and is use- or rate-independent inhibition of hKv4.3 current.

### Effect of allitridi on closed-state inactivation of hKv4.3 current

**Figure 4:**
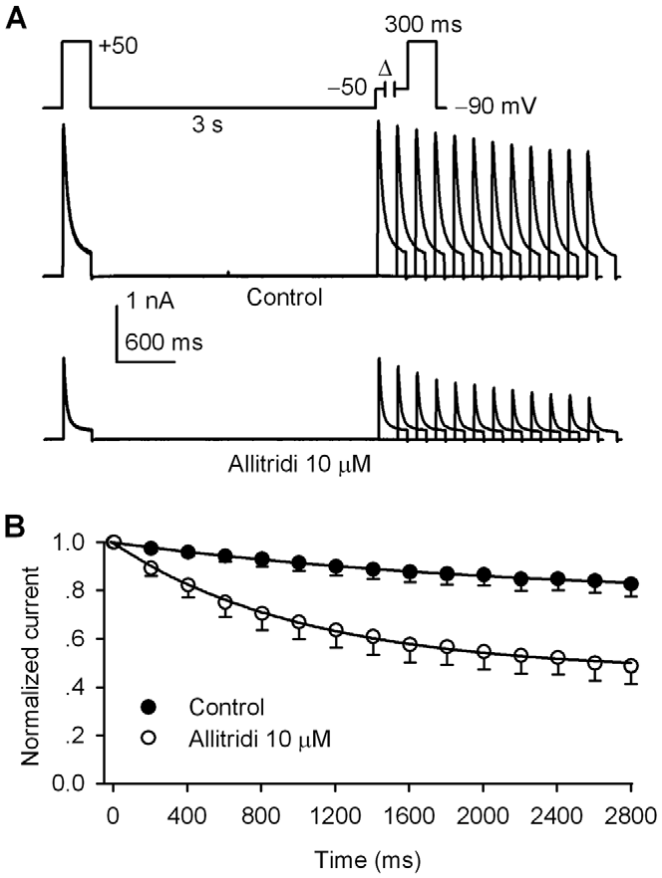
Effect of allitridi on closed-state inactivation of hKv4.3 current. **A.** The hKv4.3 current traces recorded by the voltage protocol (inset) used for determining closed-state inactivation kinetics of the channel in the absence (control) and presence of 10 µM allitridi. **B.** Mean values (n = 6) of time course of the closed-state inactivation of hKv4.3 current was fitted to a monoexpontial equation before (control) and after application of 10 µM allitridi.

The previous study reported that the steady-state inactivation of Kv4.3 channels occurs predominantly from the closed state [30], we therefore determined whether allitridi would affect the development kinetics of closed-state inactivation of hKv4.3 channels. Figure 4A illustrates the closed state inactivation current traces of hKv4.3 channels in control and after application of 10 µM allitridi. The current was recorded with a double pulses (300-ms) protocol. A progressively increasing duration of a closed state potential of −50 mV (below activation threshold) was applied for second pulse. Allitridi (10 µM) clearly accelerated the closed-state inactivation of hKv4.3 channels. The normalized second pulse current was plotted against the time duration of closed-state potential. The closed-state inactivation time course was well fitted to a mono-exponential function before and after application of 10 µM allitridi (Fig. 4B). The mean values of the inactivation time constant was 1305±25 ms in control, and 713±15 ms in 10 µM allitridi (n = 6, P<0.01 vs. control). The result suggests that allitridi significantly accelerates the kinetics of closed-state inactivation of hKv4.3channels.

### Molecular determinants of hKv4.3 channel blockade by allitridi

**Figure 5:**
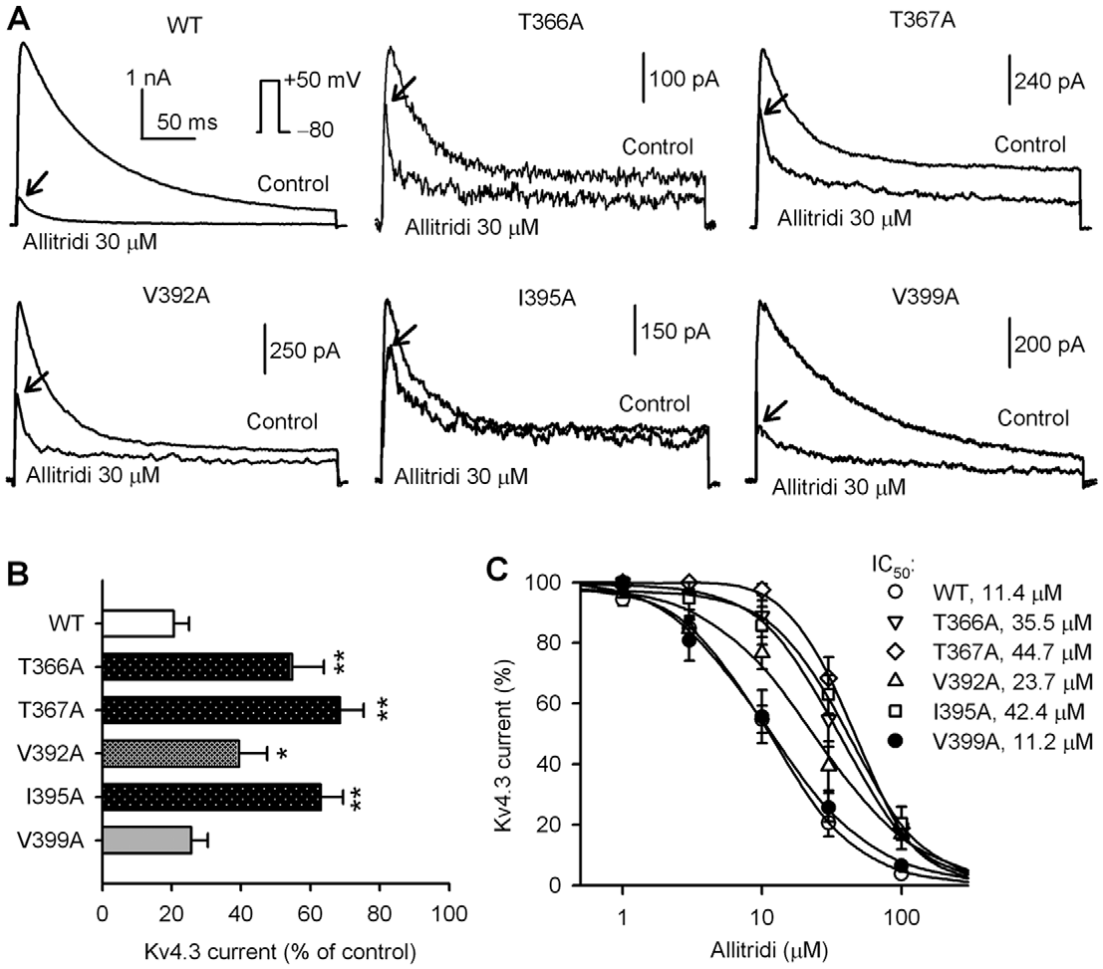
Molecular determinants of hKv4.3 channel block by allitridi. **A.** Current traces recorded in HEK 293 cells expressing WT, T366A, T367A, V392A, I395A, and V399A hKv4.3 channels, respectively, with a 300-ms voltage step to +50 mV from a holding potential of −80 mV before (control) and after 30 µM allitridi treatment for 5 min. The arrows indicate the current inhibition levels. **B.** Mean percent inhibition of WT and mutant hKv4.3 currents by 30 µM allitridi (n = 18 for control, n = 7–9 for mutants; *P<0.05, **P<0.01 vs. WT). **C.** Concentration-response relationship curves were fitted to a Hill equation to obtain the IC_50_s of allitridi for inhibiting WT and mutant hKv4.3 channels as shown in the inset (n = 7–18 for each concentration).

The molecular determinant of the block of hKv4.3 channels by allitridi was investigated using hKv4.3 mutants (see Materials and Method). These mutants are located in the pore- forming area. T366A and T367A are located in the P-loop helix, while V392A, I395A, and V399A are located in the S6 transmembrane domain. Figure 5A shows the representative current traces of wild type (WT), T366A, T367A, V392A, I395A, and V399A hKv4.3 channels activated with a 300-ms voltage step to +50 mV from a holding potential of −80 mV in the absence and presence of 30 µM allitridi. This concentration of allitridi remarkably inhibited the WT and V399A currents. A less inhibition was observed for the T366A, T367A, V392A, and I395A currents. The mean values of percentage inhibition of hKv4.3 currents are illustrated in Fig. 5B. Allitridi at 30 µM inhibited the WT hKv4.3 current by 79.4±4.4% (n = 18), T366A by 45.2±9.1% (n = 9, P<0.01 vs. WT), T367A by 31.5±6.8% (n = 8, P<0.01 vs. WT), V392A by 60.6±8.1% (n = 9, P<0.05 vs. WT), I395A by 36.9±6.4% (n = 9, P<0.01 vs. WT), and V399A by 74.3±4.3% (n = 7, P = NS vs. WT), respectively.

The concentration-dependent response to allitridi was evaluated in WT and hKv4.3 mutant currents (at +50 mV), and the concentration-response curves were fitted to a Hill equation as in Fig. 5C. The IC_50_s of allitridi in inhibiting the hKv4.3 channels were 11.4 µM for WT, 35.5 µM for T366A, 44.7 µM for T367A, 23.7 µM for V392A, 42.4 µM for I395A, and 11.2 µM for V399A, respectively. The Hill co-efficient was in between 1.2 and 1.9 in WT and mutant Kv4.3 channels. These results suggest that T366, T367, V392, and I395, but not V399, are likely the major molecular determinants of channel blocking by allitridi.

**Figure 6:**
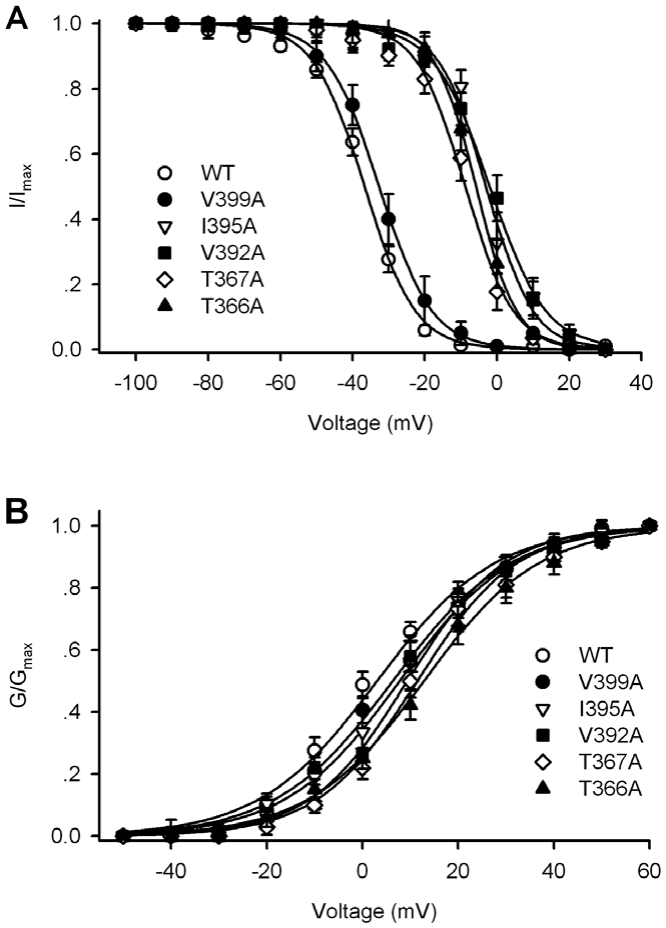
Alteration of availability and activation conductance of mutant hKv4.3 channels. **A.** Mean values of the variables of availability (I/I_max_) of WT and mutant hKv4.3 channels. **B.** Mean values of the variables of activation conductance (G/G_max_) of WT and mutant hKv4.3 channels.

To determine the relationship between the potential changes in kinetics of mutant hKv4.3 channels and the drug blocking sensitivity, we analyzed the availability (I/I_max_) and activation conductance (G/G_max_) of mutant hKv4.3 channels as in Fig. 3. Figure 6 shows the mean values of the variables of I/I_max_ and (G/G_max_) of mutant hKv4.3 channels. The variables were fitted to a Boltzmann function [29] in individual cells, and the data were summarized in Table 1. The V_1/2_ of I/I_max_ was significantly shifted to depolarization potentials in the mutants T366A, T367A, V392A, and I395A (P<0.01 vs. WT), but not V399A. Allitridi 30 µM only significantly shifted the V_1/2_ of I/I_max_ in WT hKv4.3, but not in the mutant channels (Table 1). These results suggest that the residue position is important in determining the availability of the channel and the sensitivity of hKv4.3 to block by allitridi. The V_1/2_ of G/G_max_ was slightly shifted to depolarization potentials in the mutant hKv4.3 channels, which seems not related to the sensitivity of allitridi for blocking the channel.

**Table 1:** Effects of allitridi (30 µM) on midpoint potential (V_1/2_) of availability (I/I_max_) and activation (G/G_max_) of WT and mutant hKv4.3 channels.

Type	Availability (V_1/2_, mV)			Activation (V_1/2_, mV)		
	Control	Allitridi	n	Control	Allitridi	n
WT	−36.7±1.1	−48.6±0.9##	12	3.1±1.2	−0.2±1.5	10
T366A	−5.9±1.3**	−9.2±1.1	8	6.3±1.1	4.3±1.2	7
T367A	−8.8±1.4**	−11.4±1.1	7	7.4±1.3	4.7±1.1	6
V392A	−2.1±1.2**	−5.3±1.3	6	9.3±1.6	8.1±1.3	6
I395A	−3.1±1.4**	−5.1±1.1	7	11.4±1.3*	9.1±1.5	7
V399A	−33.1±1.5	−30.7±1.4	8	12.8±1.5*	11.2±1.4	7

* P<0.05;

** P<0.01 vs. WT;

## P<0.01 vs. control.

### Effect of Allitridi on hKv1.5 Current

**Figure 7:**
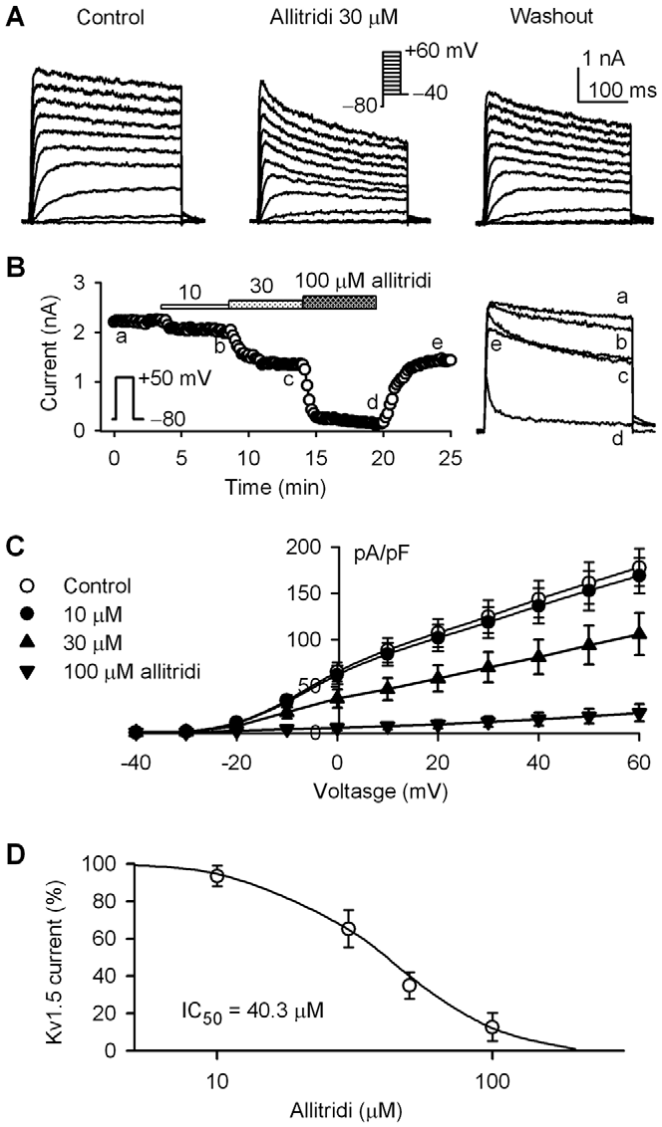
Effect of allitridi on hKv1.5 current. **A.** Voltage-dependent hKv1.5 current recorded in a representative cell with the voltage protocol as shown in the inset, in the absence and presence of 30 µM allitridi. **B.** Time-course of hKv1.5 current recorded in a typical experiment with the voltage protocol (inset) in the absence and presence of 10, 30, and 100 µM allitridi. Original current traces at corresponding time points are shown in right side of the panel. **C.** Current-voltage (*I-V*) relationships of hKv1.5 current in the absence and presence of 10, 30 and 100 µM allitridi (n = 8, P<0.05 or P<0.01 with 30 and 100 µM allitridi vs. control at −10 to +60 mV). **D.** Concentration-response relationship curve of allitridi for inhibiting hKv1.5 current was fitted to a Hill equation (n = 6–8 for each concentration).

The effect of allitridi on hKv1.5 channels expressed in HEK 293 cells was determined also with the immediately prepared working solution. Figure 7A shows the hKv1.5 current traces elicited by 300-ms voltage steps to between −40 and +60 mV from a holding potential of −80 mV in a representative cell the absence and presence of allitridi. Allitridi at 30 µM (5 min exposure) induced a significant increase of the current inactivation, a typical of open channel blockade. The inhibition was partially reversed by washout. Figure 7B illustrates the time course of hKv1.5 current recorded in a typical experiment with a 300-ms voltage step to +50 mV from a holding potential of −80 mV with accumulated application of 10, 30, and 100 µM allitridi. Allitridi at 10 µM induced a slight inhibition, while it at 30 and 100 µM remarkably suppressed the current. The inhibitory effect was partially reversed by washout.

Figure 7C displays the *I-V* relationships of mean values of hKv1.5 current in the absence and presence of allitridi. Significant inhibition of hKv1.5 current was observed with 30 and 100 µM allitridi at test potentials of −10 to +60 mV (n = 6, P<0.05 or P<0.01 vs. control). The concentration-response curve (Fig. 7D) of allitridi for inhibiting hKv1.5 current (+50 mV) was fitted to a Hill equation. The IC_50_ of allitridi for inhibiting hKv1.5 current was 40.3 µM with a Hill co-efficient of 2.1.

### Effect of Allitridi on hERG Channels

**Figure 8:**
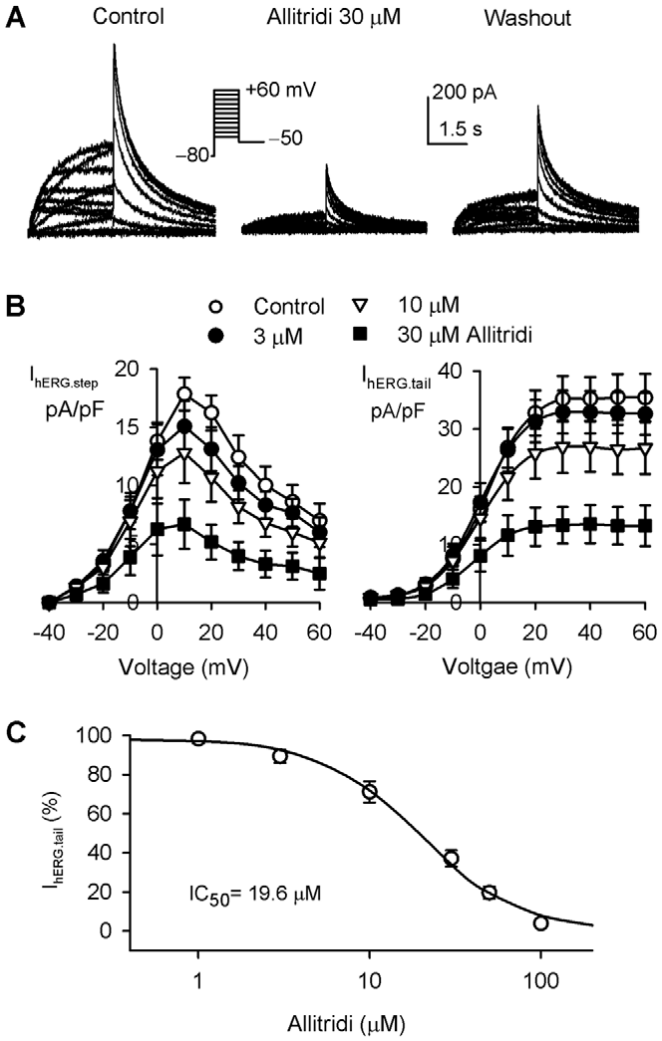
Inhibition of hERG channels by allitridi. **A.** Voltage-dependent hERG current recorded in a representative cell with the voltage protocol as shown in the inset, in the absence and presence of 30 µM allitridi. **B.** Current-voltage (*I-V*) relationships of hERG step current (I_hERG.step_) and tail current (I_hERG.tail_) in the absence and presence of 3, 10, and 30 µM allitridi (n = 7, P<0.05 or P<0.01 with 30 and 100 µM allitridi vs. control at 0 or +10 to +60 mV). **C.** Concentration-response relationship curve of allitridi inhibiting hERG tail current was fitted to a Hill equation (n = 6–8 experiments for each concentration.

The effect of allitridi on hERG channels was determined in HEK 293 cells stably expressing *KCNH2* gene [23], [25]. Figure 8A shows the voltage-dependent hERG current recorded in a typical experiment with 3-s voltage steps to between −40 and +60 mV, then to −50 mV from a holding potential of −80 mV in the absence and presence of allitridi. Allitridi at 30 µM (5 min exposure) remarkably inhibited hERG step and tail currents, and the inhibition was partially reversed by washout. Figure 8B illustrates the *I-V* relationships of hERG step current (I_hERG.step_) and tail current (I_hERG.tail_) in the absence (control) and presence of 3, 10, and 30 µM allitridi. The step current and tail current of hERG channels were significantly inhibited by 10 and 30 µM allitridi at test potentials of −20 to +60 mV (n = 7, P<0.05 or P<0.01 vs. control). The concentration-response curve of allitridi for inhibiting I_hERG.tail_ (Fig. 8C) was fitted to a Hill equation. The IC_50_ of allitridi for inhibiting I_hERG.tail_ was 19.6 µM with a Hill co-efficient of 1.5.

### Effect of Allitridi on Cardiac hKCNQ1/hKCNE1 Channels

**Figure 9:**
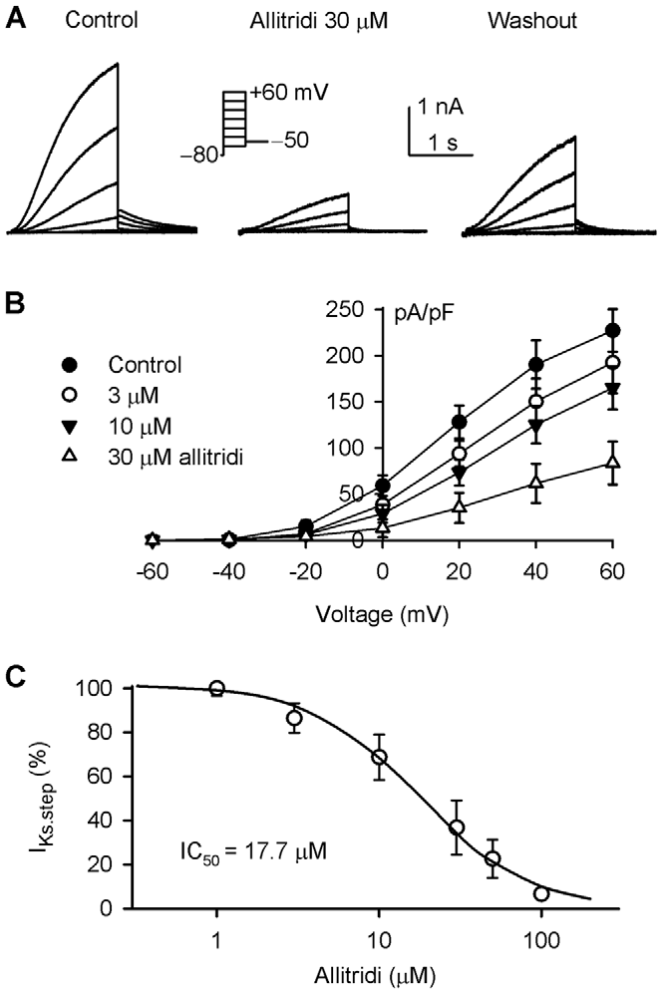
Inhibition of human cardiac I_Ks_ by allitridi. **A.** Voltage-dependent human cardiac I_Ks_ recorded in a representative HEK 293 cells expressing hKCNQ1/hKCNE1 genes with the voltage protocol as shown in the inset, in the absence and presence of 30 µM allitridi. **B.** Current-voltage (*I-V*) relationships of I_Ks_ step current in the absence and presence of 3, 10, and 30 µM allitridi (n = 8, P<0.05 or P<0.01 with 30 and 100 µM allitridi vs. control at 0 to +60 mV). **C.** Concentration-response relationship curve of allitridi inhibiting I_Ks_ was fitted to a Hill equation (n = 5–8 for each concentration).

The effect of allitridi on human cardiac I_Ks_ was determined in HEK 293 cells stably hKCNQ1/hKCNE1 [26], [31]. Figure 9A displays the voltage-dependent I_Ks_ current recorded in a representative cell with 3-s voltage steps to between −60 and +60 mV (20-mV increment), then to −50 mV from a holding potential of −80 mV in the absence and presence of allitridi. Allitridi at 30 µM (5 min exposure) remarkably inhibited the step and tail currents of I_Ks_, and the inhibition was partially reversed by washout. Figure 9B shows the *I-V* relationships of I_Ks_ step current in the absence (control) and presence of 3, 10, and 30 µM allitridi. Significant inhibition of the current was observed with 10 and 30 µM allitridi at test potentials of 0 to +60 mV (n = 6, P<0.01 vs. control). The concentration-response curve of allitridi for inhibiting I_Ks_ is illustrated in Fig. 9C, which was fitted to a Hill equation. The IC_50_ of allitridi for inhibiting human cardiac I_Ks_ was 17.7 µM with a Hill co-efficient of 1.3.

### Effect of Allitridi on hKir2.1 Current

**Figure 10:**
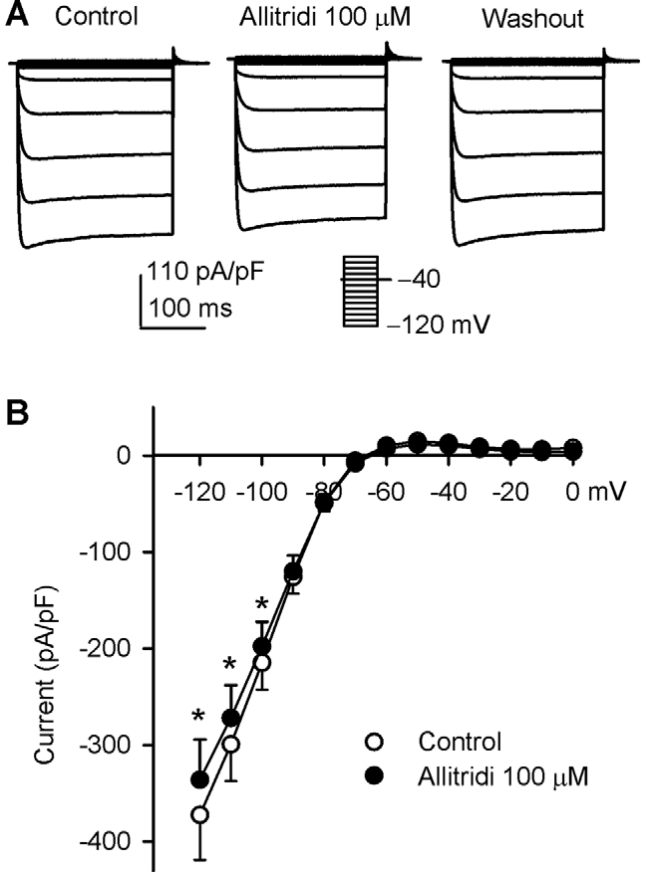
Effect of allitridi on hKir2.1 current. **A.** Voltage-dependent hKir2.1 current recorded in a representative cell with the voltage protocol as shown in the inset, in the absence and presence of 100 µM allitridi. **C.** Current-voltage (*I-V*) relationships of hKir2.1 current before (control) and after application of 100 µM allitridi (n = 6, *P<0.05 vs. control).

The effect of allitridi on hKir2.1 channels (coding the cardiac inward rectifier current I_K1_) was examined in HEK 293 cells stably expressing *KCNJ2* gene [27]. We found that allitridi had no inhibition on Kir2.1 current at 30 µM that significantly inhibited Kv4.3 Kv1.5, hERG, and I_Ks_. It induced a slight reduction of Kir2.1 current at 100 µM (Fig. 10A). The significant inhibition of Kir2.1 current was observed at test potential of −120 to −100 mV (n = 6, P<0.05 vs. control) (Fig. 10B). Kir2.1 current at −120 mV was inhibited by 9.8% with 100 µM allitridi.

## Discussion

The present study demonstrates that allitridi preferably blocks hKv4.3 channels (coding human cardiac I_to_) expressed in HEK 293 cells (IC_50_ = 11.4 µM) by binding to T366 and T367 of the P-loop helix, and V392 and I395 of the S6 domain of the channel. In addition, allitridi may also suppress other human cardiac potassium channels expressed in HEK 293 cells with a relatively weak effect, including hKv1.5 channels (coding human atrial I_Kur_, IC_50_ = 40.3 µM), hERG channels (coding human cardiac I_Kr_, IC_50_ = 19.6 µM), hKCNQ1/hKCNE1 channels (coding human cardiac I_Ks_, IC_50_ = 17.7 µM). It slightly decreases hKir2.1 channels (coding human cardiac I_K1_, by 9.8% with 100 µM allitridi). Therefore, the efficacy of allitridi for inhibiting human cardiac potassium currents is I_to_>I_Ks_>I_Kr_>I_Kur_>>I_K1_. In addition, we have shown that the inhibitory efficacy of allitridi on hKv4.3 and hKv1.5 currents is stronger than that observed previously in human atrial I_to_ and I_Kur_ [12], which suggests that the fresh preparation of the experimental working solution is important for the accurate pharmacological effect of this volatile compound. It may be non-reliable for the relative high concentration for inhibiting human atrial I_to_ observed in the previous study [12] and for blocking Kv4.3 current using a pre-prepared allitridi working solution. In addition, the previous report demonstrated that allitridi is a major biologically active volatile organosulfur compound in garlic (with a concentration of 1.1 mg/g) [32]. Whether the concentration of allitridi in garlic will have an impact on cardiac K^+^ channels function remains to be studied.

In addition to the anti-microbial effects [1], [33], [34], garlic and its constituents including allitridi were reported to have anti-cancer effects by inducing G2/M phase cell cycle arrest and apoptosis via inhibiting PI3K/Akt activation and modulating Bcl-2 family proteins [2], [35], [36]. The *in vitro* and *in vivo* studies showed that garlic and its bioactive compounds have multiple cardiovascular beneficial effects via inhibiting enzymes involved in lipid synthesis, reducing blood platelet aggregation and cholesterol, lowering blood pressure, and increasing antioxidant status [37]. Earlier studies demonstrated that garlic and its extract had anti-arrhythmic effects in ventricular tachycardia/fibrillation induced by ischemia/reperfusion in rats [38], [39], and ventricular arrhythmias induced by ouabain or isoproterenol in pigs and atrial arrhythmia in rats [11]. Rat atrial refractory period was prolonged by garlic dialysate in a concentration-dependent manner [11]. Recent studies showed that garlic extract improved defibrillation efficacy, and significantly decreased the inducibility of ventricular arrhythmia in a dose-dependent manner in a pig model [40], [41]. A more recent study has reported that allitridi has cardioprotective action against cardiac ischemia/reperfusion injury in a mouse model via releasing H_2_S which exerts a preconditioning effect [42]. However, the prolongation of cardiac effective refractory period by allitridi in isolated cardiac tissues/hearts [11], [13], [14] can not be fully interpreted by the *in vivo* H_2_S release mechanism. The blockade of multiple cardiac potassium channels by effective concentrations of allitridi observed in the present study may account for the alteration of cardiac electrophysiology and the anti-arrhythmias.

The transient outward potassium current I_to_ plays an important role in repolarization of atrial and ventricular repolarization in rodent hearts [43]. Blockade of I_to_ would prolong cardiac action potential duration and effective refractory period. Therefore, inhibition of I_to_ by allitridi may account for the increase of cardiac effective refractory period and anti-arrhythmia in rats [11], [13]. However, no I_to_ channel expression was observed in pig [15] and guinea pig [16] hearts, the anti-arrhythmic effect is likely related to the inhibition of I_Kr_ and I_Ks_ in the later species [11], [13], [14]. Previous studies demonstrated that I_Ks_ is positively regulated by Akt/PI3K kinases [44], allitridi releases H_2_S [8] and therefore induces an inhibition of Akt/PI3K [45]. Thus, the inhibition of Akt/PI3K may contribute at least in part to the decrease of I_Ks_ by allitridi.

It is well recognized that I_to_ plays an important role in the repolarization of action potentials in atrial myocytes [46], [47], [48] and also the phase 1 fast repolarization of ventricular action potentials, especially in ventricular epicardium in humans [29], [49] and in dogs [50]. Therefore, preferable blockade of I_to_/Kv4.3 and a weak inhibition of I_Kur_/Kv1.5, I_Kr_/hERG, and I_Ks_ by allitridi would prolong human atrial action potential and may be effective in anti-atrial fibrillation in humans.

It has been documented that the shift in cardiac repolarizing current due to a decrease in sodium or calcium channel currents or an increase in I_to_, I_K.ATP_, I_K.ACh_, or other outward currents may induce J-wave syndromes that involve in Brugada syndrome and early repolarization syndrome, which may trigger life-threatening arrhythmia [51], [52]. The increase of I_to_ amplitude by gain-of-function mutations in *KCND3*-encoded Kv4.3 channels is the molecular pathogenesis for the lethal arrhythmia in patients with Brugada syndrome [53]. The I_to_ blocker 4-aminopyridine restored the epicardial action potential dome, reduced both transmural and epicardial dispersion of repolarization, normalized the ST segment, and prevented phase 2 reentry and ventricular tachycardia/ventricular fibrillation in experimental Brugada syndrome [51], [54]. Allitridi has a strong inhibition of Kv4.3 current. It, as 4-aminopyridine [51], [54], is likely effective in suppressing Brugada syndrome-related arrhythmias. However, it remains to be studied in the future.

We found that the allitridi inhibited Kv4.3 channels by shifting the availability voltage to more negative potentials and accelerating the closed-state inactivation of the channel. It significantly reduced the time to peak of current activation and the time constant of Kv4.3 current inactivation. This suggests that allitridi may quickly bind the channel when they open. However, allitridi, as rosiglitazone [55], blocked the open channels of hKv4.3 in a use- or rate-independent manner. The open channel blocking effect was also observed in Kv1.5 current.

Alanine-scanning mutagenesis is a method of systematic alanine substitution and has been particularly used for the identification of functional epitopes [56]. This technique is usually used for identifying the drug binding sites of ion channel blockers [24], [57], [58]. With this technique, we demonstrated that the inhibitory efficacy of allitridi on the hKv4.3 mutants T366A and T367A at the P-loop of the pore helix was significantly reduced. This implies that allitridi may be trapped into the channel pore and block the open channel. Moreover, the mutants V392A and I395A, but not V399A, of the S6 domain exhibit a significantly reduced response to allitridi, indicating that in addition to binding to the P-helix filter, allitridi may interact with V392 and I395 of the S6 domain. Therefore, these four residues (T366, T367, V392, and I395) of the channel are likely critical for allitridi inhibition of hKv4.3 current.

Collectively, the present study demonstrates that allitridi blocks hKv4.3 channels by interacting with T366 and T367 of the P-loop helix, and V392 and I395 in the S6 domain, and also has a relatively weak inhibition of hKv1.5, hERG, and I_Ks_. These effects may count for anti-arrhythmias observed in experimental arrhythmic animal models.
